# RACIAL DIFFERENCES IN HEARING LOSS AMONG MIDDLE-AGED AND OLDER ADULTS

**DOI:** 10.1093/geroni/igad104.3548

**Published:** 2023-12-21

**Authors:** Jessica West, Sherri Smith, Matthew Dupre

**Affiliations:** Duke University, Durham, North Carolina, United States; Duke University, Durham, North Carolina, United States; Duke University, Durham, North Carolina, United States

## Abstract

Prior studies have documented how social and systemic factors contribute to racial differences in health; however, less is known about the life course and social determinants of racial differences in hearing loss (HL) among middle-aged and older adults. Evidence from U.S. population-based cohorts indicates that Black adults have better pure-tone thresholds and a 40-70% lower prevalence of HL compared to White adults. However, beyond documenting racial differences in general hearing sensitivity (i.e., degree of HL), it is unknown how the characteristics of HL (e.g., type, laterality, etc.) vary by race. Using ICD codes and the AMCLASS classification system, we will evaluate types of HL (e.g., sensorineural, conductive, and mixed HL); symmetry categories (e.g., symmetrical or asymmetrical HL); and severity categories (by classifying pure-tone average according to standard metrics: normal [-10-15 dB HL], slight [16–25 dB HL], mild [26–40 dB HL], moderate [41–55 dB HL], moderately severe [56–70 dB HL], severe [71–90 dB HL], and profound [>90 dB HL]). We will examine audiological data on 22,905 adults aged 50 and older (17.9% Black, 82.1% White) using electronic health records (EHR) and linked area-level data from a large healthcare system. We will also investigate how socioeconomic (e.g., education, income, health insurance), psychosocial (e.g., mental health), behavioral (e.g., smoking), and clinical factors (e.g., disease comorbidities) are associated with HL characteristics in White and Black adults. Findings will provide important and actionable insights into racial differences in HL.

